# Implementing interprofessional video consultations with general practitioners and psychiatrists in correctional facilities in Germany: results from a mixed-methods study

**DOI:** 10.1186/s12913-023-09592-4

**Published:** 2023-06-05

**Authors:** Miriam Giovanna Colombo, Stefanie Joos, Roland Koch

**Affiliations:** Institute for General Practice and Interprofessional Care, University Hospital Tübingen, Osianderstrasse 5, Tübingen, Baden-Württemberg 72076 Germany

**Keywords:** Telemedicine, Video consultations, Digital health, Correctional facility, General practice, Telepsychiatry, Remote consultation

## Abstract

**Background:**

Adequate health care in correctional facilities is often limited by staff shortage, which entails time-consuming consultations with physicians outside of these facilities. Video consultations (VC) have been implemented in many different health care settings and may also be useful in correctional facilities. As part of a pilot project, synchronous VC were implemented in five correctional facilities in Germany in June 2018. The aim of this study was to describe the implementation process from the providers’ perspective and to identify factors promoting or inhibiting the implementation process of VC with a focus on interprofessional collaboration between nursing staff and telemedicine physicians.

**Methods:**

As part of the mixed-methods evaluation of the pilot project, site visits to the five correctional facilities were carried out. Nursing staff from the five correctional facilities (*n=*49) and telemedicine physicians (*n=*10) were asked to participate in interviews and a questionnaire survey. Interviews were analyzed using qualitative content analysis and questionnaires were evaluated using descriptive statistical methods. The results from both data sources were integrated and discussed in the framework of Normalization Process Theory.

**Results:**

Interviews were conducted with 24.5% (*n=*12) of nursing staff and 20.0% (*n=*2) of telemedicine physicians, while questionnaires were returned by 22.5% (*n=*11) of nursing staff and 33.3% (*n=*3) of telemedicine physicians. VC with general practitioners and psychiatrists were perceived as an additional support during times when physicians were absent from the correctional facilities. Allocating telemedicine physicians to specific correctional facilities might further improve interprofessional collaboration with nursing staff during VC. Inhibiting factors comprised the lack of integrating nursing staff into the implementation process, increased workload, insufficient training and the implementation of VC at an inconvenient time.

**Conclusions:**

To summarize, VC are a promising supplement to face-to-face health care in correctional facilities despite several limitations. These might be compensated by improving interprofessional cooperation and by integrating telemedicine physicians into local health care teams.

**Supplementary Information:**

The online version contains supplementary material available at 10.1186/s12913-023-09592-4.

## Background

Like many other countries, Germany faces the challenge of ensuring adequate health care in correctional facilities (CF) [1–3]. In general, health care in German CF is provided by nursing staff as well as physicians with a background in general medicine, internal medicine or psychiatry. While some CF have sufficient medical equipment and staff at their disposal, others are poorly equipped and struggle with recruiting physicians. Patients in CF in Germany have a higher disease burden compared to the general population due to a high prevalence of psychiatric, addictive and infectious diseases [2]. Implementing telemedicine in CF has been discussed as one possible way to counterbalance physician shortage in German CF.

Especially in the United States of America, France and Australia, telemedicine interventions such as synchronous video consultations (VC) have been increasingly implemented in many different health care settings including CF over the past 20 years [4–15]. In terms of clinical outcomes, patient and provider acceptance as well as cost reduction, VC in CF have shown promising results, but potential pitfalls such as interoperability issues and lack of stakeholder involvement have also become apparent [16–19]. The transition from implementation into everyday routine practice is still one of the main challenges of pilot projects offering telemedicine services [10, 20–22]. Less research has been conducted on the factors promoting or inhibiting the process of routinely implementing VC involving telemedicine providers (nursing staff and telemedicine physicians) [16, 23]. Previous research on virtual interprofessional collaboration has shown that multiple members of a team can be responsible for developing and maintaining a good relationship between patient and telemedicine provider [23–25]. Furthermore, topics that are known to be important in interprofessional teams in general (organization of teamwork, leadership, taking up different roles) are also relevant in the context of telemedicine [23–25].

In Germany, synchronous remote health care was allowed in 2016, when the State Medical Council of Baden-Württemberg approved it within the scope of pilot projects [26]. As part of a joined pilot project of the Ministry of Justice and European Affairs and a provider of telemedicine services (A+ Videoclinic GmbH, Hamburg, Germany), VC were implemented in five CF in the federal state of Baden-Württemberg, Germany, in June 2018. The pilot project comprised synchronous VC with general practitioners (GP) and psychiatrists. The pilot project was evaluated using a mixed-methods approach [27] and the present study is based on the quantitative and qualitative data collected in the framework of this evaluation. In order to structure and integrate quantitative and qualitative results, Normalization Process Theory (NPT) was used. Originating from the field of sociology, NPT aims at understanding and explaining the process of implementing and routinely embedding a new practice into a health care setting [28], such as introducing VC in CF.

The objective of this study was to analyze and describe the process of implementing VC in CF from the perspective of nursing staff and telemedicine physicians. We aimed at identifying factors that either promoted or inhibited the process of routinely embedding VC into the health care of patients in CF with a focus on interprofessional care.

## Methods

### Setting

The pilot project was initiated by the Ministry of Justice and European Affairs of the federal state of Baden-Württemberg, Germany, and a provider of telemedicine services (A+ Videoclinic GmbH) in 2018 and comprised weekly synchronous video consultations with general practitioners and psychiatrists in five CF. Against the background of a lack of physicians willing to work in CF, the goal of the pilot project was to offer VC in addition to already existing weekly consultation hours with in-house physicians and during times when no physician was present in CF. The medical wards of the five participating CF and the telemedicine physicians taking part in the pilot project were provided with technical equipment (computer screen, laptop with video conferencing software, web camera) to conduct VC and were trained to use it by the telemedicine provider. Based on their individual needs, each CF could determine the frequency and type of VC offered (with GPs or psychiatrists only or with both). During VC, the patient and the responsible nurse sat in front of the computer screen equipped with a web camera and attended a video conference with the telemedicine physician on-call. In addition to scheduled VC hours, unplanned VC could be initiated by nursing staff outside of regular consultation hours in case of medical emergencies or when no physician was present in the CF.

### Study design and study population

A mixed-methods design was applied for the evaluation of the pilot project. It comprised site visits to the five CF, a questionnaire survey and qualitative interviews with patients, nursing staff and telemedicine physicians. Site visits were carried out first, followed by the questionnaire survey and interviews. The site visits had multiple purposes: 1^st^ to assess how health care was organized in each CF apart from the pilot project, 2^nd^ to get an overview of how the pilot project was implemented in each CF, and 3^rd^ to assess how nursing staff and patients could be contacted in the unique setting of CF, which is characterized by high security standards and limited access for outside parties. The purpose of the quantitative part of the study (questionnaire survey) was to collect data on reasons for encounter during VC and to get a first impression on how the pilot project was perceived by nursing staff, patients and telemedicine physicians. The qualitative part of the study (interviews) was conducted to get more in-depth insights into the perspectives of all parties towards the implementation process of the pilot project. Interviews and questionnaires directed at patients, who took part in VC during the pilot project, were analyzed separately and the results have been published elsewhere [27]. The focus of the present study was to describe the implementation process from the perspective of nursing staff and telemedicine physicians. Therefore, the study population of this study consisted of telemedicine physicians as well as nursing staff of the five CF, who carried out the VC on-site. NPT [28–30] was used to structure and integrate quantitative and qualitative results. Participation in any part of the evaluation study was voluntary.

### Data collection and analysis

#### Site visits

Site visits to each of the five CF were conducted by two researchers between August and November 2018. Structural data on how health care was organized and numbers of inmates as well as medical staff in each CF were collected during these site visits using a standardized checklist.

#### Survey questionnaire and quantitative analysis

Questionnaires to nursing staff and telemedicine physicians were developed based on the existing literature [20, 31]. They contained items on the process of both the implementation of the pilot project and the VC themselves, as well as questions on any previous experiences with telemedicine. Furthermore, five-point Likert scales (1=completely agree, 5=completely disagree) were used to assess whether VC were suitable for different reasons for encounter. VC were rated by the providers using grades from one to six (1=very good, 2=good, 3=satisfactory, 4=sufficient, 5=poor, 6=deficient). Additionally, socio-demographic information of the study participants was collected. Questionnaires were sent to the heads of nursing staff of each CF, who distributed them among the nursing staff (*n=*49). Questionnaires were filled out anonymously by nursing staff, collected and sent back. Questionnaires addressed to telemedicine physicians (*n=*10) were distributed by the provider of telemedicine services, filled out anonymously and returned by post or fax. The questionnaire data was analyzed using descriptive statistical methods.

#### Interviews and qualitative analysis

Two researchers conducted structured interviews with nursing staff from all five CF (*n=*12) either on-site or by telephone using an interview guideline. Interviews with telemedicine physicians (*n=*2) were conducted by telephone only. Interviews were recorded using a digital audio recording device and were subsequently transcribed. The transcripts were imported into a software for qualitative data analysis (MAXQDA) and analyzed using qualitative content analysis [32]. First, two researchers classified three of the transcripts into independent text units that were then grouped together into meaningful categories. Second, these categories were again subsumed under different main categories. The resulting system of main and subcategories (coding frame) was then applied to the remaining transcripts (inductive approach) [33]. Any discrepancies between the researchers were resolved by discussion. Finally, the main and subcategories of the coding frame were screened for topics that were repeatedly mentioned by several interview partners. This included reviewing the quotes from each category for any recurring topics and labeling them accordingly.

#### Integrating quantitative and qualitative results using NPT

The authors of NPT focus on understanding the individual and communal work that needs to be done in order to embed and sustain a newly implemented practice in the framework of complex interventions [28, 29], such as offering VC in CF. NPT has been applied prospectively as well as retrospectively in several studies using data originating from quantitative and qualitative as well as mixed-methods studies [11, 30]. May and colleagues defined four mechanisms (coherence, cognitive participation, collective action and reflexive monitoring) that provide a framework to identify patterns in the implementation process that are either beneficial or harmful for a successful transition into routine practice. Further details on the four mechanisms of NPT can be found in Additional file 1.

To apply NPT to our data and to integrate the results from quantitative and qualitative analyses, we assigned the recurring topics identified through content analysis and the quantitative results from survey questionnaires to the different mechanisms and components of NPT. Only those items and topics that proved to be related to the implementation process of VC were considered. Based on this combination of NPT and quantitative as well as qualitative data, we subsequently identified factors that enabled or interfered with a successful long-term implementation of VC in CF.

### Ethics approval and consent to participate

Written informed consent was obtained from all study participants prior to conducting the interviews. Interviews were recorded using a digital audio recording device, transcribed and pseudonymized. Audio files were deleted following the transcription. Questionnaires were filled out anonymously. Both analogue and digital data will be deleted and destroyed ten years after the evaluation study has ended.

Ethics approval for the evaluation of the pilot project was obtained from the ethics committee of both the State Medical Council of Baden-Württemberg (reference number: F-2018-054) and the University Hospital of Tübingen (reference number: 728/2018BO1). The study was conducted in accordance with the Declaration of Helsinki [34].

## Results

A total of 14 interviews were conducted with nursing staff (*n=*12, 24.5%) and telemedicine physicians (*n=*2, response rate 20.0%). Eleven questionnaires (22.5%) were returned by nursing staff and three (33.3%) by telemedicine physicians. Characteristics of each CF as well as numbers of returned questionnaires and interviews are presented in Table 1. Interviews were conducted with nursing staff from all five CF whereas questionnaires were returned from four facilities. The duration of the interviews ranged from 30 to 60 minutes.

**Table 1:** Characteristics of the participating correctional facilities and numbers of questionnaires and interviews [27]

	**Inmates**			**Nursing staff**			**Telemedicine physicians**		
**CF**	**Age group**	**Sex**	**n**^**a**^	**n**^**a**^	**Interviews** **n (%)**^**b**^	**Questionnaires, n (%)**^**b**^	**n**	**Interviews** **n (%)**	**Questionnaires, n (%)**
1	Adults	M	350	9	1 (11.1)	1 (11.1)	10	2 (20.0)	3 (33.3)
2	Adults and adolescents	F	350	6	3 (50.0)	4 (66.7)			
3	Adolescents	M	395	9	4 (44.4)	3 (33.3)			
4^c^	Adults	M	52	3	3 (100.0)	3 (100.0)			
5	Adults	M	772	22	1 (4.6)	0			
Total			1.919	49	12 (24.5)	11 (22.5)			

*CF* Correctional facility, *F* Female, *M* Male

^a^Numbers of inmates and nursing staff were inquired during site visits to the correctional facilities in 2018

^b^Percentage of the number of nursing staff in each correctional facility

^c^Social therapy facility

Interviews with nursing staff and telemedicine physicians were analyzed using content analysis. The resulting system of main and subcategories can be found in Table 2. Topics that occurred repeatedly in these main and subcategories were allocated to the NPT subcomponents. The results structured by the NPT mechanisms and components are shown in Table 3.

**Table 2:** Main and subcategories identified through content analysis

**Main category**	**Subcategory 1**	**Subcategory 2**
**Implementing VC in CF**	Pilot project	-
	Infrastructure	-
	Integrating VC in existing work	Choosing patients for VC
	Initial training for employees	Continuing training
		Support
	Extending VC to other CF	-
**Health care in CF**	Access to health care in CF	-
	Evaluating health care in CF	-
	Communication with patients in CF	-
	Safety	-
	Tasks of medical staff	Mission
		Changes due to VC
**Medical staff in CF**	Job situation	-
	Cooperation of medical staff	Continuity
	Dependence on management/hierarchy	-
	Reputation/appreciation	-
	Requirements for medical staff	Medical training
		Individual work experience
		Special requirements for VC
**Process of VC**	Safety aspects	-
	Preparation and follow-up	-
	Reasons for encounter and diagnoses	-
	Communication with patients during VC	Interpreter
		Physical presence of conversation partners
	Result of VC	-
**Evaluation of VC**	Image and sound quality	-
	Limitations and opportunities of VC	VC without patients
		Use of VC
		Capacity of VC
	Consequences for medical staff	Decision-making support
		Salary
	Comparison with regular health care in CF	-
	Interface	-
	Suggestions for improvement and future upgrades	Technical upgrades
		Organizing VC
		Training
		One-on-one VC
		Additional fields of application
**Case reports**	-	-

*CF* Correctional facility, *VC* video consultation

**Table 3:** Results from content analysis in the context of mechanisms and components of Normalization Process Theory

**A. Coherence**	**B. Cognitive participation**	**C. Collective action**	**D. Reflexive monitoring**
**A.1 Differentiation**	**B.1 Initiation**	**C.1 Interactional Workability**	**D.1 Systematization**
Health care in CF apart from VC • According to nursing staff, the health care of patients is sufficient compared to health care outside of CF • Reduced access to health care for inmates during the night and on weekends • Nursing staff in CF work autonomously. This involves decision-making processes without physician’s support. Taking responsibility for first-line medical decisions is part of nursing staff’s professional identity	• Medical staff has to be present during VC to prevent damage to VC equipment	• No additional time was allocated for VC • Additional workload caused by implementing VC: VC has to be conducted in addition to regular workload • Timely documentation of VC by telemedicine physicians was necessary • Lower amount of sick days (patients), since VC provided the possibility to schedule a consultation immediately instead of having to wait for regular consultation hours of physicians in CF • Duplication of work: Patients had to consult the physician in the CF in addition to VC	• VC were suitable for times when no physician is present in the CF • VC were not suitable for emergencies • VC were suitable for night shifts or as background services • VC should be implemented at an appropriate time (avoiding vacation season, staff shortage, renovation in CF) • Nursing staff had a positive impression of telemedicine physicians and describe them as open-minded and communicative • Collaborating with telemedicine physicians was easier if telemedicine physicians spoke in a calm, neutral and professional way during VC • It negatively impacted their cooperation when telemedicine physicians questioned the medical treatments administered by physicians in CF • The possibility to use interpreters during VC was seen was an advantage • Image and sound artifacts had an impact on the quality of VC
VC compared to regular health care • Staff situation and resources are limited in CF and provide little room for VC • VC are more time-intensive in terms of preparation and documentation of the consultations, VC are less time efficient • Many processes in CF are paper based and thus require physical presence of a physician (for signatures etc). Signatures cannot be provided during VC yet			
**A.2 Individual specification**	**B.2 Legitimation**	**C.2 Relational integration**	**D.2 Individual appraisal**
• On an individual level, members of the nursing staff were curious of the new health care service/VC • Nursing staff was sceptical towards the new technology/VC • Telemedicine physicians had different experiences in working in CF, ranging from many years of experience to no experience at all • Computer skills were beneficial for the acceptance of the new health care service • Telemedicine physicians should be trained in medical treatment of patients with addictive diseases • Most nursing staff were already experienced in consultations via telephone • Medical staff had a strong positioning as self-sufficient problem managers. To a large extent, they made first-line medical decisions on their own	• Nursing staff perceived the implementation process as a top-down-process	• Flexibility/adaptability required from telemedicine physicians • Building mutual trust is essential for a successful long-term cooperation between nursing staff and telemedicine physicians • Establishing a trustful cooperation between nursing staff and telemedicine physicians takes time • Nursing staff motivated patients to participate in VC • Establishing a trustful relationship between telemedicine physicians and patients takes time	• Instructions provided by colleagues (nursing staff) were not sufficient as initial training • Enough time and a quiet environment was essential to practice and learn how to conduct VC • VC can become a routine practice if practiced regularly (learning by doing) • Discrepancies in terms of technical knowledge of nursing staff and members of the technical support hotline of the telemedicine provider • Telemedicine physicians were satisfied with the remuneration for VC
**A.3 Communal specification**	**B.3 Enrolment**	**C.3 Contextual integration**	**D.3 Communal appraisal**
• VC should be implemented at an appropriate time (avoiding vacation season, staff shortage, renovation in CF) • VC was considered meaningful in non-critical situations, e.g. sick leave or substitution, when enough time was available • VC were described as an alternative when physicians were absent (e.g sick leave of physicians, on weekends or during night time) • VC without a patient was considered useful by nursing staff • Data protection and medical ethics had to be guaranteed during VC		• Recurring time delays (due to technical or organizational issues) during VC negatively impacted the implementation process • The weekday, time of day and available nursing staff (for VC) need to be planned and agreed upon in advance • To ensure continuity, telemedicine physicians need to be appointed to specific CF	• Physicians in CF cannot be replaced by VC • VC should be implemented at an appropriate time • VC were suitable for evaluating the inability to work and for issuing sick leaves • VC were suitable for admission examinations only to a limited extent • It was not possible to treat the same number of patients in the same amount of time using VC compared to regular consultation hours • To ensure sufficient training, initial training courses should be offered for new colleagues (nursing staff) at the beginning of their employment • Insufficient internet connection and frequent interruptions of image and sound obstructed VC
**A.4 Internalization**	**B.4 Activation**	**C.4 Skillset workability**	**D.4 Reconfiguration**
• Nursing staff acknowledged that VC can provide additional support for their own medical decisions when needed • VC were perceived as unnecessary if telemedicine physicians had to refer patients to existing health care services in CF • VC can prevent transfers of inmates to nearby hospital or physician outside of CF • Nursing staff feel supported in their decision-making by VC	• Technical equipment had to be installed. • A suitable room for VC had to be found • Instruction and training of nursing staff was needed • Training should to be provided by specialists of the VC company • When equipment was missing it was quickly provided by the telemedicine provider • In case any technical problems occurred, they were quickly solved by the telemedicine provider • The quality of the internet connection had an impact on the quality of VC	• Weekly VC hours need to be planned in advance: patients need to be selected for VC while considering the medical specialty of the available telemedicine physician • To facilitate the conversation between telemedicine physicians and patients, nursing staff acted as medical “interpreters” during/after VC • A clear and neutral wording facilitated the communication with the patients • Communicating with patients and nursing staff was the most important tool during VC, since telemedicine physicians were not able to physically examine the patients themselves	• VC should be used in a needs-oriented and flexible way • The possibility for patients to talk to telemedicine physicians without nursing staff in certain cases (e.g. psychiatric cases) should be provided • Additional medical specialties for VC: Dermatology, urology, rheumatology, ear nose throat specialists • Organize VC in collaboration with other federal states, e.g. using a shared database of telemedicine physicians (if the pilot project is implemented in other federal states) • Image and sound quality should be improved
		New tasks/responsibilities of nursing staff: • In addition to their regular tasks, nursing staff took over physicians’ tasks during VC (e.g.auscultation, palpation) • Nursing staff acted as intermediaries, mediators and facilitators between telemedicine physicians and patients during VC • Nursing staff passed on information to telemedicine physicians during VC: medical history of the patients, specific information about the CF (e.g.available medication, possible medical procedures) • Organizing, preparing and following-up VC	Extension of VC/additional equipment: • Digital, electronic stethoscope and otoscope • High-resolution camera, dermatoscope • Offer the possibility to upload documents into the software in order for telemedicine physicians to access them directly (physician’s letter, diagnostic findings, medical imaging) • Interpreter (already exists) • Camera for specially secured detention areas in CF, body camera for nursing staff • Provide telemedicine physicians with access to electronic files of the CF (interface) • Offer training with new equipment

*CF* Correctional facility, *VC* Video consultation

A table with exemplary quotes from interviews and questionnaires (translated from German into English) for the main and subcomponents can be found in the supplementary material (see Additional file 2). Since we applied a mixed-methods study design, both quantitative and qualitative results are presented below (direct quotes from interviews: *N=*nursing staff, *P*=telemedicine physician).

### Coherence

The nursing staff compared the VC with current health care for patients in the CF and were asked about their expectations towards the pilot project in the early stage of the implementation process. In general, health care in the CF was described as sufficient, but nurses also referred to it as being limited on the weekends and during the night. They described themselves as being used to both working autonomously as well as assisting the physicians in the CF.

*N: "When we're on our own, we try to do as much as we can…what we are capable of doing thanks to our training […]. And when we realize that we need a physician, we either transport the inmate to a physician outside of prison or we call an ambulance in case of an emergency."* (TASCAM 61:7-7)

VC were perceived as more time-consuming mainly due to the expected amount of documentation. As most of the documentation in the CF relies on paperwork, telemedicine physicians could not provide signatures (e.g. for prescriptions of medications), which limited the usefulness of VC according to nursing staff. At the beginning of the implementation process of the pilot project, telemedicine physicians had no access to the medical records of the patients, which also impeded the process of sharing medical information with the telemedicine physicians. Furthermore, a stable relationship between patient and physician was deemed especially valuable in the setting of CF. Therefore, concerns were raised about VC with changing telemedicine physicians, as planned in the VC concept of the telemedicine service provider. Additionally, nursing staff preferred telemedicine physicians, who were already experienced in working with patients in CF and in treating addictive disorders.

Some members of the nursing staff were curious of the new technology, whereas others were skeptical towards it. Nursing staff who had already conducted telephone consultations before and who were experienced in working on a computer were more receptive towards VC.

*N: "It was very stressful for me…because I'm kind of an…I don't want to say "anti-talent", but I don't do much with computers. Only the absolutely necessary things. I don't use a computer at home, neither for online banking nor for writing letters. I only taught myself the essentials that I need at work. Especially with the electronic medical records and so on. That was very stressful for me. However, as soon as I had worked with it a couple of times, I was able to manage it, 'learning by doing' of course. I don't think it's a big problem. Of course, if you didn't receive proper training and a password...that was a stressful situation for me."* (TASCAM 72:19)

Telemedicine physicians had more experience with VC and were familiar with it before the pilot project started.

P: “I am very pleased, but I already had experiences in the navy…the navy already uses telemedicine for quite some time now and I did the training to become a navy physisican and they already practiced telemedicine back then. It wasn't new for me and I knew it from other countries." (TASCAM 89 physician:36)

The nursing staff agreed that it should be avoided to implement VC in times of staff shortage or when reconstructive measures are carried out that involve the medical ward of the CF. Furthermore, VC were considered useful when no physicians were available in the CF and for non-emergency medical services. Nursing staff had a positive attitude towards using VC to seek medical advice from telemedicine physicians as second opinions and as specialist input, respectively.

*N: "I've also experienced the following: it is not always necessary to put the patient in front of the monitor. Sometimes you can manage it without them being there. I see it like an on-call physician. We didn't have that all these years."* (TASCAM 61:39)

*N: "Especially when it comes to times when our regular physician is not available… sometimes one is insecure concerning some issues and it is encouraging…you have a better feeling knowing that someone is there, who you can talk to. You are also more self-confident towards the prisoners. In my opinion it is a very positive support for us nurses."* (TASCAM 61:35)

In the interviews, nursing staff were of the opinion that transfers to hospitals or physicians outside of the CF could be avoided using VC. In the survey questionnaires only 45.5% of nursing staff, but all telemedicine physicians agreed that transfers could be reduced using VC (see Additional file 3, Table 1, item C11).

*N: "It's beneficial when it comes to the medication of a new prisoner, who suffers from drug or alcohol withdrawals. It's not necessary to take the patient to a hospital. We can avoid staff shortage and stress. Those are important in prison. Because it's a whole different story if someone can go there by himself. It's different in prison. Enough staff and so on are needed.”* (TASCAM 72:17)

In contrast, conducting VC was perceived as unnecessary if telemedicine physicians had to refer patients to physicians in the CF instead of treating them themselves. According to the nursing staff and in line with NPT, VC had to provide an additional benefit for the health care of the patients in order to be routinely implemented. Nursing staff also acknowledged that VC provided them with additional security and relief when it came to their medical decision-making.

*N: "It can be a relief from a medial point of view. Not so much from an organizational or prison-related point of view. But if you really need medical advice it can be helpful to make a decision."* (TASCAM 74:95)

### Cognitive participation

Preparatory work was needed to implement the pilot project. This included the search for an appropriate space to conduct the VC as well as establishing a digital infrastructure such as installing a data landline or network cables. The preparatory work also included the training of nursing staff in the CF.

From the nurses’ perspective, a suitable room had to be found for conducting VC, before the technical equipment could be installed. In this context, nursing staff noted that a member of the nursing team always had to be present during VC due to safety reasons. Not all CF could provide a separate space to conduct VC and nurses had to use the existing treatment room, blocking it for other patients.

*N: "I always sit next to the patient during the conversation. Also, because there is lots of equipment that should not get lost."* (TASCAM 61:45)

To familiarize themselves with the technical equipment, nursing staff and telemedicine physicians received initial training. According to the questionnaire survey, 72.7% of nursing staff and all telemedicine physicians (100%) received training on how to use the technical equipment at the beginning of the implementation process (see Additional file 3, item F1). 62.5% of nursing staff considered the initial training as “good”, while all telemedicine physicians gave it a “very good” rating (see Additional file 3, Table 2, item F1). The majority of nurses (82.8%) invested between one and four hours of preparation in the initial stages of the pilot project, while telemedicine physicians (100%) needed three to four hours of preparation (see Additional file 3, Table 2, item F7).

Furthermore, if there was any technical material missing or if any technical problems occurred, it was important for the nursing staff to know that it could be quickly supplied or solved, respectively.

Little initiative was taken to actively involve nursing staff in the implementation process, according to the interviews. Even though the head nurses of each CF were included into the implementation process, the nursing staff had the impression that the pilot project was implemented using a top-down-approach and that they had only little or no say in it.

*N: "It is a telemedical consultation hour that was imposed on us in the framework of a pilot project and we now have to continue it."* (TASCAM 73:47)

### Collective action

The implementation of VC affected both the regular work of the nurses and created a new setting of interprofessional teamwork. The nursing staff reported that even though the implementation of VC resulted in an increased workload, no additional time was allocated for it during regular working hours. Furthermore, it was considered more time-consuming, when patients had to consult another physician in the CF after the VC. In the survey questionnaire, both the workload needed to prepare and to follow-up the VC were described as (rather) extensive by more than 70% and 60% of nursing staff, respectively. For the telemedicine physicians, the amount of work to follow-up the consultations exceeded the amount of preparatory work (see Additional file 3, Table 3, items B2, B3). More than 90% of the respondents of the questionnaire survey (rather) agreed that the VC increased the total workload for nursing staff (see Additional file 3, Table 3, item C13). Only 36% of respondents believed that the VC could be incorporated in the daily routine of the CF without any problems (see Additional file 3, Table 3, item C14).

*N: "At one point, we refused to do the video consultations in addition to our regular work. We made use of it during night shifts and on weekends; it made a lot of sense to us. Or when I know that our physician is sick or on vacation. We set a date for one or two days, we offer consultation hours, but first we always filter those people, who don't really need a physician in our opinion."* (TASCAM 77:27)

Asked whether the implementation of the pilot project had changed any in-house organizational, technical or logistical processes, nursing staff gave contrasting answers. On the one hand, they reported that more patients could be treated without delay and outside of the regular consultation hours. They also noticed a decreased number of patients, who called in sick since the pilot project was implemented. On the other hand, they expressed the need for more staff due to the increased workload. The majority felt that they were insufficiently staffed to offer both regular consultations and VC.

*N: "I have to say that it [the pilot project] was introduced at the worst time...I believe it was last year in August. It was some kind of cut: now we have to do it. All nurses were totally exhausted. We had one physician, but he was only part-time employed. We had so much to do, it was summer time and vacation time - and on top of that the telemedicine project was introduced. I mean, everyone who worked during that time was rather old and not very experienced in working on the computer. It was a very stressful situation for all of us."* (TASCAM 72:37)

Recurring time delays due to technical or scheduling errors were attributed to the implementation process, thus negatively affecting its acceptance. To avoid such delays, scheduling of VC and staff planning needed to be carried out in advance, which further increased the workload for the head nurses.

N: "*In general, we use a special laptop with internet access for this purpose to log into the system of the provider. Then we have to create a profile for the patient so that the physician, who sits on the other end, has access to the data of the patient and has the possibility to document everything. It’s a cumbersome process, but I understand the necessity. However, one day we had a lot to do and lots of patients, who were planned to be seen by the telemedicine physician. And then you have to create a profile for 7 to 8 patients at the same time. That takes up a lot of time that could be spent doing other things.”* (TASCAM 74:21)

During the interviews, several interview partners (nurses and telemedicine physicians) discussed the relatively new setting of interprofessional team work in the framework of VC and how VC affected the interaction between CF staff and external physicians. Asked about a general evaluation of their cooperation, both telemedicine physicians and the majority of nursing staff gave the rating “very good” or “good” in the survey (see Additional file 3, Table 3, item D1). According to nursing staff, building mutual trust was essential for a successful cooperation between telemedicine physicians and nursing staff or patients, respectively. Furthermore, telemedicine physicians were described by nursing staff as being open-minded and communicative, which also facilitated their cooperation.

*N: "You can communicate well with both of them [telemedicine physicians]. The communication takes place eye to eye - even though that sounds stupid. You can talk to them. They react to the problems and take their time and also show interest. I have a positive impression of both of them."* (TASCAM 63:51)

From the perspective of telemedicine physicians, it was important to get to know the nurses and the extent of their medical knowledge to be able to delegate tasks such as physical examinations during VC.

*P: "If there are colleagues on the other side, who I know and I know what they are able to do...do you know what I mean? But if there are colleagues, who I know less and I cannot judge their abilities, I concentrate on the patient and I'm a bit limited in terms of what I can or cannot delegate. But if I work as an emergency physician and I have to work with other people, it is the same thing. But if a psychiatrist does the consultations for a while, it will get better. It is only like this in the beginning and it will get better."* (TASCAM 89 physician:63)

Their cooperation was perceived to be easier by nursing staff, if telemedicine physicians were flexible when it came to scheduling consultation hours. Telemedicine physicians had to promptly provide the nursing staff with their report of the consultation after VC, since nursing staff were responsible for transferring the information into the documentation system of the CF. The nursing staff considered VC more time-consuming, if telemedicine physicians did not provide their documentation of the consultation in time.

*N: "The physicians have time until the next day. In depends on how much time they have. Sometimes it doesn't take much time and the report arrives after half an hour. But it can also take until the next day."* (TASCAM 62:14)

When the pilot project was first implemented in the CF, nursing staff undertook several new tasks. Prior to carrying out VC, nursing staff had to schedule consultation hours and select suitable patients. During VC they acted as intermediaries and mediators between telemedicine physicians and patients. They carried out tasks normally performed by physicians, such as auscultating or palpating and reporting on their findings.

*N: "In general, I am the link [between physician and patient]. The physician is far away and only sees the patient on the monitor and the patient only sees the physician on the monitor. When it comes to things like measuring blood pressure, general vital signs ....then I have to do it. Therefore, I'm the link, the executive power."* (TASCAM 74:45)

They also provided the telemedicine physicians with information about the patients (e.g. medical history) but also about the specific circumstances of health care in the CF (e.g. available medication). Both nurses and telemedicine physicians attributed vital knowledge to the patient’s history, their social situation and any hidden agenda the patient might have . They emphasized the importance of this information, which was provided by the nurses either before or after the VC.

### Reflexive monitoring

In general, VC were deemed to be suitable for different types of CF. Nursing staff considered them to be useful during times when no physicians were present in the CF, especially during the night and as an on-call service. More than 90% of respondents agreed that VC should be continued to be offered outside of regular consultation hours (see Additional file 3, Table 4, item A10). In contrast, VC were not perceived as being useful for emergencies according to nursing staff, since they preferred to directly contact emergency services outside of the CF.

*N: "When someone comes to us with a life-threatening disease, I wouldn't consult the telemedicine physician, but set off an alarm in order to call for help. So that emergency services and an emergency physician are notified and the emergency kit is brought to the scene.* (TASCAM 62:61)

It was important for nursing staff to have enough time to practice how to use VC. Some nursing staff was not able to participate in the initial training at the beginning of the pilot project and was, therefore, introduced to VC by their colleagues. However, the training provided by other nursing staff was seen as less effective than the training provided by the telemedicine provider. Over 60% of respondents indicated that they would have preferred more training by the telemedicine provider at the beginning of the pilot project (see Additional file 3, Table 4, item F4). Asked how often VC trainings should be offered, 30% of nursing staff were of the opinion that one training at the beginning of the pilot project was sufficient, while others suggested at least semi-annual trainings (see Additional file 3, Table 4, item F6). In contrast, telemedicine physicians were satisfied with the amount of training they received at the beginning of the pilot project (see Additional file 3, Table 4, items F4, F6).

Furthermore, nursing staff had the possibility to call the technical support service of the telemedicine provider in case they experienced technical problems during VC. In this context, nursing staff found it frustrating when misunderstandings occurred due to their own lack of technical knowledge.

Nursing staff and telemedicine physicians agreed that the existing health care provided by physicians in the CF could not be replaced by VC. The main reason was that it was not possible to treat the same number of patients in the same amount of time using VC compared to regular consultation hours. While VC were suitable for issuing sick leaves, their use for admission examinations was deemed limited (see Additonal file 3 Table 4, item C4). Furthermore, insufficient internet connections and frequently occurring image and sound interruptions caused further time delay.

*N: "Of course, the were some communication problems in the beginning. But the sound was always an issue. The sound was always bumpy and it never worked out. It was problematic in the beginning. I'm not sure why it happened, I'm not a technician, but it was not very satisfying." (TASCAM 77:41)*

Based on their experiences with VC, nursing staff proposed possible improvements of the pilot project. First, they emphasized that VC should be tailored to the needs of each CF. This included adjusting the frequency of VC hours according to the respective staff situation and to the demand for additional health care services. Second, it was proposed to offer the possibility for patients to consult a physician using VC without members of the nursing staff being present.

P: "It could be an advantage also for psychiatric consultations - but not for all of them. For some specific patients it could be an advantage, if they were able to discuss their problems or diseases or the crime they committed…or in the psychiatric ward, when they have a history of abuse or something similar or they have problems with officials or the prison director and they don't want to communicate it to any of the officials. Otherwise that would be problem." (TASCAM 89 physician:56)

Third, extending the pilot project to telemedicine physicians from other medical specialties, e.g. dermatology, was suggested by nursing staff. Fourth, to facilitate the documentation process for nursing staff, it was suggested that telemedicine physicians should get direct access to the electronic patient data of each CF. In fact, this suggestion was implemented at the end of the pilot project. Finally, potential additional equipment such as specialized cameras or digital stethoscopes is listed in Table 3. In total, 54.5% of nursing staff gave the VC the rating “good” and 45.5% the rating “satisfactory”, whereas 66.7% of telemedicine physicians rated them as “very good” (see Additional file 3, Table 4, item G1).

## Discussion

In this mixed-methods study, the process of implementing VC in CF was described from the perspectives of nursing staff and telemedicine physicians. Using NPT, factors that might have promoted or harmed the implementation process are discussed below with a focus on how interprofessional collaboration might be optimized during the implementation process.

### Coherence

Video consultations proved to be a valuable addition to health care in CF, especially during times when no physicians were present (during the night and on weekends). This finding was in line with previous studies evaluating the implementation of VC in this setting [4, 5, 12, 13]. In contrast to previous studies [16], our results showed that nurses did not fear a loss of autonomy through VC, but rather felt that the implementation of VC was not tailored to their needs. While past research identified scheduled VC to be an enabler of the implementation process, CF nurses in our study preferred a more flexible schedule. They wanted to choose whether VC was indicated based on their needs and the current staff situation. Based on their individual preferences and experiences, most nurses were open towards the new technology, but criticized the timing of the implementation of the pilot project, which has been identified as an important factor in previous research [15].

### Cognitive participation

In terms of the preparatory work that was needed to implement VC in CF, our results revealed substantial gaps in the implementation process. First, appropriate training at the beginning of the implementation phase was deemed necessary by nursing staff. From their point of view, the implementation lacked repeated training sessions, especially when new nursing staff was introduced to VC. Continuous training is a known prerequisite for projects implementing telemedicine and has been identified in previous studies on telemedicine services, also in CF [14].

Another issue that was mentioned in the interview was a lack of stakeholder involvement. The importance of including relevant stakeholders has been emphasized in previous research [13]. In line with studies on previous telemedicine projects, we identified a lack of involving relevant stakeholders in the pilot project early on [6, 18, 19]. Nursing staff especially felt that neither the individual circumstances of the CF, nor administrative processes were sufficiently considered, which are known barriers in the implementation process according to Deslich and colleagues [8]. In contrast, telemedicine physicians felt adequately prepared for conducting VC. These differing perceptions of the implementation process shows that even though both professions were separately prepared to conduct VC, less attention was paid to how their cooperation could facilitated.

The implementation process was mainly perceived as a top-down-approach by nursing staff. This could have been avoided, for example, by organizing a visit of a representative of the Ministry of Justice and European Affairs of the federal state of Baden-Württemberg to inform and involve staff at the beginning of the pilot project [16]. In addition, offering joint interprofessional workshops for telemedicine physicians and nurses at the beginning of the pilot project could facilitate their collaboration.

Involving local champions [15], mainly head nurses in the CF, positively contributed to the implementation process. While the presence of local champions is a known enabler in pilot projects [15], our results showed that this new role substantially increased their own workload during the implementation process.

The preparatory work in the implementation process included finding an appropriate space for conducting VC, which was successful in most of the CF. However, some CF did not have additional space and had to convert other areas normally used for medical examination to fit the technical equipment. As a result, regular in-person consultations and VC could not be conducted at the same time and nursing staff were of the impression that the VC replaced valuable direct contact with the patients, which is a known barriers in the implementation process of VC [16]. Network infrastructure and dedicated rooms for VC were often lacking, which in turn added to the workload of preparing and documenting individual VC. While studies from the early 2000s identified network bandwidth or video quality as limiting factors [15, 18], this study found barriers in the organizational and managerial processes. This shows that digital technological advances not necessarily improve work processes.

### Collectice action

In line with a study by Deslich et al. [8], our results showed that nursing staff played several roles during VC. First, they acted as mediators and facilitators between patients and telemedicine physicians. For example, they informed the telemedicine physicians about any substance abuse or other relevant medical issues of the patient that were not explicitly mentioned by the patient during the consultation. Second, they also acted as an interface between telemedicine physician and patients by performing physical examinations and reporting their findings back to the telemedicine physicians, who were not able to perform these important medical tasks themselves. Third, nurses took over additional administrative tasks, such as selecting patients for VC, preparing the consultations and documenting them afterwards. Both groups acknowledged the new roles of nursing staff and assessed the collaboration as successful.

In line with previous research, our results showed that interprofessional collaboration in the framework of telemedicine is complex [6, 24, 25]. The additional tasks of nursing staff added to the complexity of interprofessional collaboration and could have been avoided if the implementation process had been optimized beforehand. This could have been done, for example, by permanently assigning specific telemedicine physicians to each CF, by building interprofessional teams to select patients for VC [23] and by providing telemedicine physicians with access to the patient data management system of the CF early in the implementation process [17]. In addition, it could be helpful to interlink health care provided through VC with the regular health care in the CF, for example by including cases from VC in existing interdisciplinary case conferences of the CF. This could also promote efficient working relationships outside of telemedical health care [16].

Since telemedicine physicians only act as external consultants, they do not become members of the team [23]. While best efforts were made by both sides (nurses and telemedicine physicians) to deliver high quality care during VC, the complexity and added dimensions of the implementation process impeded a sustainable formation of interprofessional teams [23–25]. In line with existing literature, our results showed that clearly defined roles and team building efforts are as important in telemedicine as in traditional health care [9, 11, 16, 23–25].

### Reflexive monitoring

Reflecting on the first months of the pilot project, it became clear that VC were not applicable for all participating CF in the same way. While some CF used VC with both GPs and psychiatrists, others preferred one over the other. In addition, some facilities were only willing to use VC outside of weekly consultations hours, while others had the time and staffing resources to offer VC hours twice a week. Thus, before implementing VC in other CF, it is recommended to assess and identify the current demand and to tailor VC hours to the needs and prerequisites of each facility.

The present study confirms some known perceived aspects of VC in CF settings. Adequate health care can be provided using VC, especially in case of minor, non-acute complaints while maintaining a safe environment for the patient and complying with safety measures in the CF [8, 12, 16]. While VC increased access to health care, when there was no physician available in CF, the implmentation of VC parallel to face-to-face health care comes at the cost of additional workload for nursing staff (e.g. due to the need to select patients for VC and additional documentation work).

Even though many suggestions were made on how to extend the pilot project in the future, for example by offering VC with other medical specialties or by providing additional medical equipment, they did not appear to be crucial factors for the future routine application of VC. The same applied for technical issues. Image or sound disturbances during VC only rarely occurred during the implementation phase of the pilot project. The nursing staff was under the impression that VC with telemedicine physicians supported them in their medical decisions. However, in contrast to telemedicine physicians, nurses were undecided whether transfers to physicians or hospitals outside of CF could be avoided and replaced by VC.

### Strengths and limitations

This is the first evaluation study of a pilot project implementing telemedicine in CF in Germany. The majority of previous studies that evaluated a newly implemented telemedicine project in CF collected data on provider satisfaction. However, they were limited to general evaluations and only reported whether providers were overall satisfied with the newly implemented practice or the technology used [12, 20]. Our goal was to provide a more detailed description and understanding of the implementation process from the providers perspective. By using a mixed-methods design, we were able to collect data from all participating CF and to illustrate the implementation process from different angles. Additionally, applying NPT to our data allowed us to identify critical gaps and issues in the implementation process in a structured way.

This study has several limitations. First, selection bias might have occurred since both nursing staff and telemedicine physicians were not chosen at random for the interviews and questionnaires. Since the majority of the data for this study originated from interviews with and questionnaires to nursing staff, our result mainly mirror their experiences with VC. Due to the small number of telemedicine physicians, who participated in this study, our results might not cover all aspects of the physicians’ perspective. Second, since questionnaires were filled out anonymously, we cannot rule out that study participants took part in an interview and also completed a questionnaire. Thus, experiences and opinions of those who did both might be overrepresented in this study. Third, due to a larger amount of data, the qualitative data likely had a greater impact on our conclusions than the quantitative data. However, the questionnaire survey helped us to quantify aspects of the implementation process, such as the amount of work needed to prepare the consultations and the perceived usefulness of VC for different reasons of encounter. Finally, NPT does not consider any cognitive components of behavior that might affect whether someone routinely adopts a new practice or not [29]. Additionally, we applied NPT retrospectively and were therefore limited to the available data. This became especially apparent when the quantitative results were assigned to the mechanisms and components of NPT. The majority of quantitative results related to the component of reflexive monitoring while for the other components, quantitative results were underrepresented compared to qualitative results. Even though prospectively applying NPT might have been superior, our approach is in line with previous evaluation studies using NPT [11, 30].

## Conclusions

Currently, VC are a promising supplement to face-to-face primary care in CF despite several limitations, such as an overlap with existing health care services and increased workload for nursing staff. These can be compensated by improving interprofessional cooperation and by integrating telemedicine physicians in CF with local health care teams. To establish efficient virtual interprofessional teamwork [24], mutual recognition, flexibility, allocated time for team meetings and development must be considered during the implementation process both for nursing staff and telemedicine physicians [16, 23, 25]. Ideally, stakeholders are included in the process even before the implementation commences in order to prevent negative attitudes towards it, to avoid initial difficulties and to make use of synergetic effects early on.

## Supplementary Information

**Additional file 1.** Mechanisms and components of Normalization Process Theory.

**Additional file 2.** Mechanisms and components of Normalization Process Theory illustrated with quotes from interviews and questionnaires.

**Additional file 3.** Quantitative results from questionnaires to nursing staff and telemedicine physicians.

## Data Availability

The authors confirm that the majority of data supporting the findings of this study are available within the article and its supplementary materials. Additional data (interview transcripts) are available from the corresponding author, upon reasonable request.

## Abbreviations

CF: Correctional facility
GP: General practitioner
N: Nursing staff
NPT: Normalization process theory
P: Telemedicine physician
VC: Video consultation
