# Supplementary material for: Implementing interprofessional video consultations with general practitioners and psychiatrists in correctional facilities in Germany: results from a mixed-methods study
Source: BMC Health Serv Res. 2023 Jun 5;23:578. doi: 10.1186/s12913-023-09592-4 (PMC10242990; doi:10.1186/s12913-023-09592-4)
Supplement: Supplementary file 1 — Additional file 1. Mechanisms and components of Normalization Process Theory. [file 12913_2023_9592_MOESM1_ESM.docx]

**Additional file 1**

**Normalization Process Theory**

May and colleagues defined four mechanisms (*coherence, cognitive participation, collective action and reflexive monitoring*) that provide a framework to identify patterns in the implementation process that are either beneficial or harmful for a successful transition into routine practice (1). First, *coherence* represents the notion that actors need to attribute meaning and utility to the new practice (1). This can be achieved for example by identifying characteristics that set VC apart from conventional medical care in correctional facilities. Second, c*ognitive participation* refers to the (preparatory) work that needs to be done in order to carry out VC, including the active integration of actors who are supposed to put them into practice (1). Third, *collective action* is defined as the work of embedding VC and deals with the way VC might change the interactions between the actors as well as between them and the organizational context (1). Finally, *reflexive monitoring* refers to the ongoing process of evaluating and reconfiguring VC (1). The four mechanisms are further subdivided into two components (*immediate* and *organizing work*), each containing two further subcomponents (see Table 1).

According to the authors of NPT, implementing a new practice not only requires the actual work of the actors applying it, but also involves their investment into it. As shown in Table 1, each mechanism includes a specific investment. The investment of *meaning* refers to the significance that is attributed to the new practice by the actors applying it in the respective social context. *Commitment* implies the need for dedication and engagement shown by the actors. Furthermore, an *effort* needs to be made to uphold a new practice. Finally, *comprehension* refers to a shared understanding of the new practice that the actors develop during the implementation process (1).

**Table 1: Elements of Normalization Process Theory (1)**

|  |  | **Mechanisms** | | | |
| --- | --- | --- | --- | --- | --- |
|  |  | **Coherence** | **Cognitive participation** | **Collective action** | **Reflexive monitoring** |
| **(Sub-) Components** | **Immediate work** | Differentiation | Initiation | Interactional workability | Systematization |
|  |  | Individual specification | Legitimation | Relational integration | Individual appraisal |
|  | **Organizing work** | Communal specification | Enrolment | Contextual integration | Communal appraisal |
|  |  | Internalization | Activation | Skill set workability | Reconfiguration |
|  | **Investments** | Meaning | Commitment | Effort | Comprehension |

**References**

1. May C, Finch T. Implementing, Embedding, and Integrating Practices: An Outline of Normalization Process Theory. Sociology. 2009;43(3):535-54.
