# Supplementary material for: Implementing interprofessional video consultations with general practitioners and psychiatrists in correctional facilities in Germany: results from a mixed-methods study
Source: BMC Health Serv Res. 2023 Jun 5;23:578. doi: 10.1186/s12913-023-09592-4 (PMC10242990; doi:10.1186/s12913-023-09592-4)
Supplement: Supplementary file 3 — Additional file 3. Quantitative results from questionnaires to nursing staff and telemedicine physicians. [file 12913_2023_9592_MOESM3_ESM.docx]

**Additional file 3: Quantitative results from questionnaires to nursing staff and telemedicine physicians**

**Table 1: Coherence**

|  | | **Nursing staff** N=11 | **Telemedicine physicians** N=3 |
| --- | --- | --- | --- |
|  |  | n (%) | n (%) |
| C11 The number of transports to physicians outside the CF can be reduced. | Agree | 1 (9.1) | 2 (66.7) |
|  | Somewhat agree | 4 (36.4) | 1 (33.3) |
|  | Undecided | 3 (27.3) | 0 |
|  | Somewhat diagree | 2 (18.2) | 0 |
|  | Disagree | 1 (9.1) | 0 |

CF, correctional facility.

**Table 2: Cognitive participation**

|  | | **Nursing staff** N=11 | **Telemedicine physicians** N=3 |
| --- | --- | --- | --- |
|  |  | n (%) | n (%) |
| F1 Did you receive training at the beginning of the implementation? | Yes | 8 (72.7) | 3 (100) |
| F2 If yes, how would you rate the initial training? | Very good | 0 | 3 (100) |
|  | Good | 5 (62.5) | 0 |
|  | Satisfactory | 2 (25.0) | 0 |
|  | Sufficient | 0 | 0 |
|  | Poor | 0 | 0 |
|  | Deficient | 0 | 0 |
|  | Missing value | 1 (12.5) | 0 |
| F7 How many working hours did you invest into the preparation of the VC^a^? | <1 hour | 1 (9.1) | 0 |
|  | 1-2 hours | 5 (45.5) | 0 |
|  | 3-4 hours | 3 (27.3) | 3 (100) |
|  | 5-6 hours | 1 (9.1) | 0 |
|  | 7-8 hours | 0 | 0 |
|  | >8 hours | 1 (9.1) | 0 |
| F8 How did you perceive the amount of work that went into preparing the VC? | Extensive | 3 (27.3) | 0 |
|  | Somewhat extensive | 4 (36.4) | 1 (33.3) |
|  | Reasonable | 3 (27.3) | 1 (33.3) |
|  | Somewhat low | 1 (9.1) | 1 (33.3) |
|  | Low | 0 | 0 |

VC, video consultation.

^a^ Training, learning how to use technical equipment and software.

**Table 3: Collective action**

| **Collective action** | | **Nursing staff** N=11 | **Telemedicine physicians** N=3 |
| --- | --- | --- | --- |
|  |  | n (%) | n (%) |
| B2 How do you rate the amount of work that is needed to prepare a VC? | Extensive | 3 (27.3) | 0 |
|  | Somewhat extensive | 5 (45.5) | 0 |
|  | Reasonable | 3 (27.3) | 0 |
|  | Somewhat low | 0 | 2 (66.7) |
|  | Low | 0 | 1 (33.3) |
| B4 How do you rate the amount of work that is needed to follow up a VC? | Extensive | 2 (18.2) | 0 |
|  | Somewhat extensive | 5 (45.5) | 1 (33.3) |
|  | Reasonable | 3 (27.3) | 2 (66.7) |
|  | Somewhat low | 1 (9.1) | 0 |
|  | Low | 0 | 0 |
| B5 Did the implementation of VC change (organizational) processes within the CF? | Yes | 8 (72.7) | n/a |
| C13 The VC increased the total workload of nursing staff in the CF. | Agree | 6 (54.5) | n/a |
|  | Somewhat agree | 4 (36.4) |  |
|  | Undecided | 1 (9.1) |  |
|  | Somewhat diagree | 0 |  |
|  | Disagree | 0 |  |
| C14 The VC could be integrated into the daily routine of the CF | Agree | 1 (9.1) | n/a |
|  | Somewhat agree | 3 (27.3) |  |
|  | Undecided | 0 |  |
|  | Somewhat diagree | 4 (36.4) |  |
|  | Disagree | 3 (27.3) |  |
| D1 How do you rate the cooperation with the telemedicine physicians/nurses? | Very good | 3 (27.3) | 2 (66.7) |
|  | Good | 7 (63.6) | 1 (33.3) |
|  | Satisfactory | 1 (9.1) | 0 |
|  | Sufficient | 0 | 0 |
|  | Poor | 0 | 0 |
|  | Deficient | 0 | 0 |

CF, correctional facility; n/a, not applicable; VC, video consultation.

**Table 4: Reflexive monitoring**

|  | | **Nursing staff** N=11 | **Telemedicine physicians** N=3 |
| --- | --- | --- | --- |
|  |  | n (%) | n (%) |
| A10 Do you think that it is useful to continue offering VC outside of regular consultation hours? | Yes | 10 (90.9) | n/a |
| C1 VC can cover the different reasons to seek medical treatment that occur in the CF | Agree | 0 | 2 (66.7) |
|  | Somewhat agree | 5 (45.5) | 1 (33.3) |
|  | Undecided | 4 (36.4) | 0 |
|  | Somewhat diagree | 2 (18.2) | 0 |
|  | Disagree | 0 | 0 |
| C2 Patient with chronic conditions can be adequately treated using VC in CF. | Agree | 1 (9.1) | 1 (33.3) |
|  | Somewhat agree | 2 (18.2) | 2 (66.7) |
|  | Undecided | 4 (36.4) | 0 |
|  | Somewhat diagree | 3 (27.3) | 0 |
|  | Disagree | 1 (9.1) | 0 |
| C3 VC are useful for consultations that concern the patient’s medication (prescription, adjusting the dosage) | Agree | 2 (18.2) | 2 (66.7) |
|  | Somewhat agree | 4 (36.4) | 1 (33.3) |
|  | Undecided | 4 (36.4) | 0 |
|  | Somewhat diagree | 1 (9.1) | 0 |
|  | Disagree | 0 | 0 |
| C4 VC are useful for admission examinations. | Agree | 0 | 1 (33.3) |
|  | Somewhat agree | 2 (18,2) | 1 (33.3) |
|  | Undecided | 2 (18,2) | 1 (33.3) |
|  | Somewhat diagree | 4 (36,4) | 0 |
|  | Disagree | 2 (18,2) | 0 |
|  | Missing value | 1 (9.1) | 0 |
| C5 VC improve the health care of patients in CF. | Agree | 3 (27.3) | 2 (66.7) |
|  | Somewhat agree | 4 (36.4) | 1 (33.3) |
|  | Undecided | 4 (36.4) | 0 |
|  | Somewhat diagree | 0 | 0 |
|  | Disagree | 0 | 0 |
| C6 Compared to regular health care in CF, patients are treated more promptly using VC **on weekends** | Agree | 2 (18.2) | n/a |
|  | Somewhat agree | 6 (54.5) |  |
|  | Undecided | 0 |  |
|  | Somewhat diagree | 2 (18.2) |  |
|  | Disagree | 0 |  |
|  | Missing value | 1 (9.1) |  |

**Table 4: continued**

|  | | **Nursing staff** N=11 | **Telemedicine physicians** N=3 |
| --- | --- | --- | --- |
|  |  | n (%) | n (%) |
| C7 Compared to regular health care in CF, patients are treated more promptly using VC **during the night** | Agree | 2 (18.2) | n/a |
|  | Somewhat agree | 6 (54.5) |  |
|  | Undecided | 0 |  |
|  | Somewhat diagree | 2 (18.2) |  |
|  | Disagree | 0 |  |
|  | Missing value | 1 (9.1) |  |
| C8 Compared to regular health care in CF, patients are treated more promptly using VC **during the week** | Agree | 1 (9.1) | n/a |
|  | Somewhat agree | 2 (18.2) |  |
|  | Undecided | 5 (45.5) |  |
|  | Somewhat diagree | 2 (18.2) |  |
|  | Disagree | 1 (9.1) |  |
| C9 Compared to regular health care in CF, patients are treated more promptly using VC **when no physician is present in the CF** | Agree | 4 (36.4) | n/a |
|  | Somewhat agree | 5 (45.5) |  |
|  | Undecided | 1 (9.1) |  |
|  | Somewhat diagree | 1 (9.1) |  |
|  | Disagree | 0 |  |
| C10 Compared to regular health care in CF, patients are treated more promptly using VC **when a physician is present in the CF** | Agree | 0 | n/a |
|  | Somewhat agree | 0 |  |
|  | Undecided | 1 (9.1) |  |
|  | Somewhat diagree | 6 (54.5) |  |
|  | Disagree | 4 (36.4) |  |
| F4 Would you prefer to receive additional training by the telemedicine provide? | Yes | 7 (63.6) | 0 |
| F6 How frequently should the training be offered? | Once at the beginning | 3 (27.3) | 2 (67.7) |
|  | Every 2 months | 1 (9.1) | 0 |
|  | Quarterly | 2 (18.2) | 0 |
|  | Semi-annually | 3 (27.3) | 0 |
|  | Annually | 1 (9.1) | 0 |
|  | Missing value | 1 (9.1) | 1 (33.3) |
| G1 How do you rate the VC in general? | Very good | 0 | 2 (66.7) |
|  | Good | 6 (54.5) | 1 (33.3) |
|  | Satisfactory | 5 (45.5) | 0 |
|  | Sufficient | 0 | 0 |
|  | Poor | 0 | 0 |
|  | Deficient | 0 | 0 |

CF, correctional facility; n/a, not applicable; VC, video consultation.
